# Diabetes Mellitus Is Still a Strong Predictor of Periprocedural Outcomes of Primary Percutaneous Coronary Interventions in Patients Presenting with ST-Segment Elevation Myocardial Infarction (from the ORPKI Polish National Registry)

**DOI:** 10.3390/jcm11216284

**Published:** 2022-10-25

**Authors:** Artur Dziewierz, Barbara Zdzierak, Krzysztof P. Malinowski, Zbigniew Siudak, Wojciech Zasada, Tomasz Tokarek, Michał Zabojszcz, Magdalena Dolecka-Ślusarczyk, Dariusz Dudek, Stanisław Bartuś, Andrzej Surdacki, Tomasz Rakowski

**Affiliations:** 1 2nd Department of Cardiology, Institute of Cardiology, Jagiellonian University Medical College, 2 Jakubowskiego St., 30-688 Krakow, Poland; 2 Department of Cardiology and Cardiovascular Interventions, University Hospital, 2 Jakubowskiego St., 30-688 Krakow, Poland; 3 Department of Bioinformatics and Telemedicine, Jagiellonian University Medical College, 31-008 Krakow, Poland; 4 Digital Medicine & Robotics Center, Jagiellonian University Medical College, 31-008 Krakow, Poland; 5 Collegium Medicum, Jan Kochanowski University, 25-369 Kielce, Poland; 6 Center for Invasive Cardiology, Electrotherapy and Angiology, 33-300 Nowy Sacz, Poland; 7 Center for Innovative Medical Education, Jagiellonian University Medical College, 30-688 Krakow, Poland

**Keywords:** myocardial infarction, diabetes mellitus, angioplasty, complications, registry

## Abstract

The impact of diabetes mellitus (DM) on outcomes of patients with ST-segment elevation myocardial infarction (STEMI) undergoing primary percutaneous coronary intervention (PCI) was confirmed by several studies. However, it is unclear whether this effect is still present in large groups of unselected patients undergoing up-to-date treatment. Thus, we sought to assess the impact of DM on periprocedural outcomes of primary PCI in STEMI using data from the Polish National Registry of PCI. Data on 150,782 STEMI patients undergoing primary PCI were collected. Of them, 26,360 (17.5%) patients had DM. Patients with DM were higher-risk individuals who experienced longer reperfusion delays and were less likely to have closed infarct-related artery at baseline (TIMI 0 + 1 flow: 73.2% vs. 72.0%; *p* < 0.0001) and achieve optimal reperfusion after PCI (TIMI 3 flow: 91.8% vs. 88.5%; *p* < 0.0001). The periprocedural mortality (1.1% vs. 1.9%; *p* < 0.0001) was higher in patients with DM and DM was identified as an independent predictor of periprocedural death. In conclusion, despite continuous progress in STEMI treatment, DM remains a strong predictor of periprocedural mortality. However, this detrimental effect of DM may be partially explained by the overall higher risk profile of diabetic patients.

## 1. Introduction

Early reperfusion with primary percutaneous coronary intervention (PCI) is a life-saving treatment for ST-segment elevation myocardial infarction (STEMI) patients [1]. Although several predictors of worse outcomes after primary PCI for STEMI, including diabetes mellitus (DM), were identified [2,3,4,5,6,7]. The impact of DM on outcomes of STEMI patients might be justified by the overall higher risk profile and more complex coronary artery disease of diabetic patients [2,3,4,5]. In addition, enhanced platelet adhesion, activation, and aggregation are observed in patients with DM, leading to an increased risk of periprocedural complications [8,9,10,11]. However, the influence of these factors might be mitigated by new treatment strategies and modern pharmacotherapy of STEMI [12,13,14]. Whether the impact of DM on outcomes of STEMI patients undergoing primary PCI is still present in unselected patients undergoing up-to-date treatment is unclear. Thus, we sought to assess the impact of DM on periprocedural outcomes of primary PCI using data from the Polish National Registry of PCI (ORPKI).

## 2. Materials and Methods

**Figure 1:**
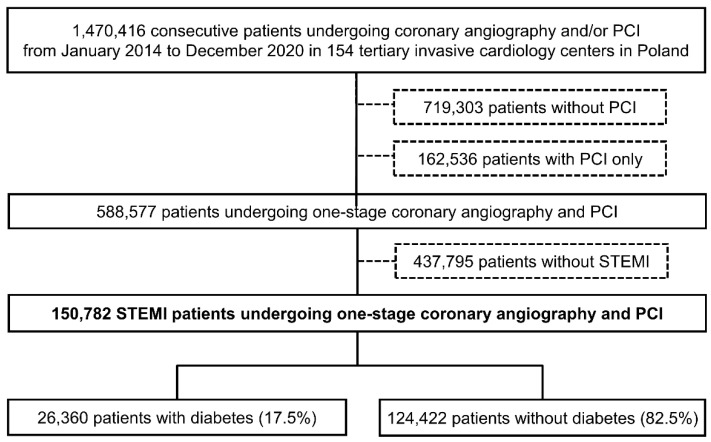
Study flow chart. Abbreviations: PCI = percutaneous coronary intervention; STEMI = ST-segment elevation myocardial infarction.

The ORPKI is a national registry operated by the Jagiellonian University Medical College in Krakow that collects data on all percutaneous procedures in interventional cardiology performed in Poland [15,16,17,18,19]. Data on all consecutive patients without strict inclusion/exclusion criteria were collected from January 2014 to December 2020 in 154 invasive cardiology centers. For this analysis, data on 150,782 consecutive patients presenting with acute STEMI who had undergone one-stage coronary angiography and primary PCI were retrieved from the database (Figure 1). The patients were then stratified according to the presence of DM. Patients with a history of DM treated with insulin, oral hypoglycemic agents, or diet were classified as diabetic patients. No data concerning the type of DM (type 1 or 2), duration of symptoms, and type and dose of oral hypoglycemic drugs were collected. STEMI diagnosis was established by treating physicians according to established guidelines. All angiographies/PCIs were carried out according to current medical standards. The decision regarding concomitant pharmacotherapy and procedural technique was the operator’s choice. The primary endpoint was all-cause periprocedural (in cathlab) mortality. In addition, data on other periprocedural complications, including stroke, cardiac arrest, coronary artery perforation, dissection, no-reflow, allergic reaction, and puncture site bleeding were collected. The assessment of complications, as well as grading of epicardial flow with the Thrombolysis in Myocardial Infarction (TIMI) scale before and after PCI was based on the operator’s judgment.

Continuous variables are presented as median (interquartile range) and were compared using the Mann–Whitney U test. Categorical variables are presented as percentages and were compared with Pearson’s chi-squared test or Fisher’s exact test if 20% of cells had an expected count <5. Trends were analyzed using the Cochran–Armitage test. The relationship between TIMI grade 0 to 1 at baseline, TIMI 3 after PCI, periprocedural death, no-reflow, and any complications and DM was analyzed using a logistic regression model and presented as odds ratios (OR) with 95% confidence intervals (CI). All patient demographics, medical history, and procedural details were considered potential predictors. Then, the final models were constructed using the stepwise approach with minimization of Bayesian Information Criterion as the target with DM lock in the model. Then, additional models with the inclusion of all clinically relevant variables were constructed to limit the risk of excluding important confounders during such procedures. The level of statistical significance was set at *p* < 0.05. All analyses were done with JMP^®^, Version 15.1.0 (SAS Institute Inc., Cary, NC, USA) and R, version 4.0.4 (R Foundation for Statistical Computing, Vienna, Austria, 2021) with the package: ‘rms’, version 6.0-1.

## 3. Results

**Figure 2:**
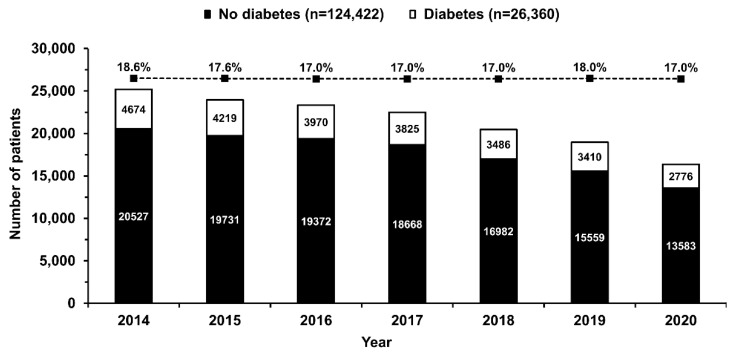
Number of patients with (empty bars) and without (solid bars) diabetes mellitus undergoing primary percutaneous coronary intervention in ST-segment elevation myocardial infarction included in the subsequent years of the registry. The percentage of patients with diabetes mellitus decreased slightly from 2014 to 2020 (dotted line, *p* < 0.0001 for trend). Values are presented as numbers or percentages.

Between 2014 and 2020, data from 150,782 STEMI patients undergoing one-stage coronary angiography and primary PCI were collected in the ORPKI Registry. Of the 150,782, 26,360 (17.5%) patients had DM. The decline in the overall number of STEMI patients treated during the study period and the percentage of patients with DM from 18.6% in 2014 to 17.0% in 2020 was observed (Figure 2). Patients with DM were older and more frequently women. In addition, these were higher-risk individuals with a higher prevalence of arterial hypertension, previous MI, previous coronary revascularization, and numerous comorbidities (Table 1). Despite the less frequent out-of-hospital cardiac arrest, patients with DM were more likely to be in cardiogenic shock (Killip class IV) on admission. Also, they experienced longer delays to first medical contact and PCI than patients without DM (Table 1).

**Figure 3:**
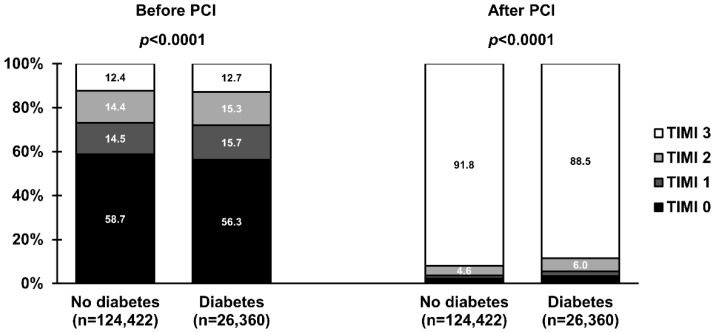
Frequencies of Thrombolysis in Myocardial Infarction (TIMI) grade 0 to 3 flow before and after primary percutaneous coronary intervention (PCI) in ST-segment elevation myocardial infarction stratified by the presence of diabetes mellitus. Values are presented as percentages.

The use of femoral access was more common in patients with than without DM (Table 2). Interestingly, the left anterior descending artery was identified more frequently as the infarct-related artery in patients with DM. In addition, more complex anatomy, including higher prevalence of multivessel disease and bifurcations, and the need to implant more stents during PCI was noted in patients with DM, which resulted in higher radiation and contrast media load. Regarding the epicardial flow, patients with DM were less likely to have a closed infarct-related artery at baseline (TIMI grade 0 to 1 flow: 73.2% vs. 72.0%; *p* < 0.0001) and achieve optimal reperfusion after the procedure (TIMI grade 3 flow: 91.8% vs. 88.5%; *p* < 0.0001) than patients without DM (Figure 3). In the multivariate analysis, DM was not identified as an independent predictor of TIMI grade 0 to 1 flow at baseline (Table 3). However, it remained significant in the adjustment model with an adjusted OR (95% CI) of 0.95 (0.91–0.99); *p* = 0.030. In contrast, DM was independently associated with the lack of successful reperfusion (lower chance for TIMI grade 3 flow after PCI, Table 3) with an adjusted OR (95% CI) of 0.86 (0.80–0.92); *p* < 0.001.

**Figure 4:**
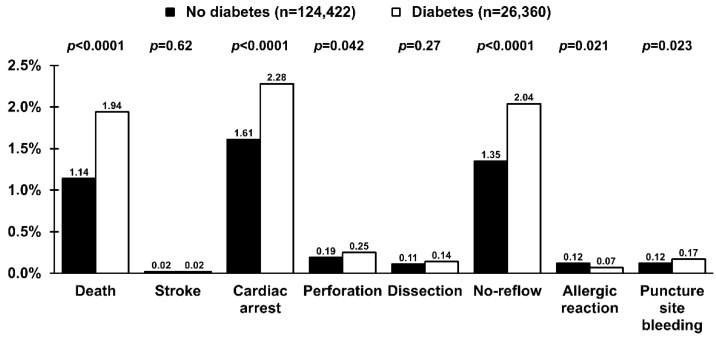
Periprocedural complications in patients with (empty bars) and without (solid bars) diabetes mellitus undergoing primary percutaneous coronary intervention in ST-segment elevation myocardial infarction. Values are presented as percentages.

There were 1927 (1.3%) all-cause periprocedural deaths. The risk of death (1.1% vs. 1.9%; *p* < 0.0001), as well as cardiac arrest, coronary artery perforation, no-reflow, allergic reaction, and puncture site bleeding, was higher in patients with DM (Figure 4). Several independent predictors of periprocedural death, including DM, were identified (Table 3) with an adjusted OR (95% CI) of 1.32 (1.11–1.57); *p* = 0.002. However, cardiogenic shock (Killip class IV) was the strongest one. Although the risk of no-reflow was higher in patients with DM, this relationship was no longer significant after adjustment for covariates (Table 3, adjusted OR (95% CI) 1.07 (0.92–1.23); *p* = 0.38). On the other hand, DM was an independent predictor of any periprocedural complication (Table 3). However, only with a trend towards increased periprocedural complications in the adjustment model with adjusted OR (95% CI) of 1.10 (1.00–1.19); *p* = 0.06). 

## 4. Discussion

Our main finding is that despite great advances in STEMI treatment, DM remains an important predictor of periprocedural complications, including periprocedural death. This finding aligns with recent studies confirming higher short- and long-term morbidity and mortality in patients with DM presenting with STEMI [11,20].

On average, the presence of DM doubles the risk of cardiovascular diseases associated with atherosclerosis, including acute MI [21]. Therefore, the coexistence of DM in patients with STEMI is quite common [11]. In our cohort, 17.5% of STEMI patients had a history of DM. This value conforms to the results of other registries. However, the reported frequencies may vary across studies and strongly depend on the enrolled population and the definitions used [2,22,23,24,25,26]. For instance, a recent report from the Polish Registry of Acute Coronary Syndromes (PL-ACS) reported DM in 28.4% of patients with ACS [23]. This value is much higher than the one reported in our study, as data on DM diagnosed during hospital stay in the ORPKI registry were not available. In addition, the prevalence of DM in STEMI patients might be lower than that observed in the general population of patients with ACS [22,25]. The observed decrease in the number of patients with DM was accompanied by a decrease in the overall number of patients with STEMI undergoing PCI. This gradual reduction in STEMI patients was confirmed in other primary-PCI networks. The explanations are the positive influence of pharmacotherapy and other primary prevention measures, and better access to invasive diagnosis of coronary artery disease, which may decrease the likelihood of acute presentation of MI. A recent, sharp decrease in the number of STEMI patients treated with primary PCI was strongly associated with the COVID-19 pandemic [19,27,28]. This was accompanied by an increase in ischemia time and worse outcomes [18,27,28]. Importantly, this negative impact of the COVID-19 pandemic was observed in both patients with and without DM [24].

In our study, patients with DM were more likely to have a patent infarct-related artery on the baseline angiogram. However, this difference in the infarct-related patency has vanished after adjustment. Furthermore, previous studies that focused on this issue did not confirm the relationship between diabetic status and patency of infarct-related artery before PCI [29,30,31,32]. On the other hand, DM was strongly associated with less complete reperfusion at the epicardial level and a lower chance of achieving TIMI grade 3 flow after PCI. This finding is in line with the results of several studies and might be justified by the presence of more complex and diffused coronary artery disease [4,33]. Also, DM may lead to a prothrombotic state characterized by platelet hypersensitivity, hypofibrinolysis, and coagulation factor disorders [8]. It may result in a higher thrombus load within the infarct-related artery and a higher risk of angiographic complications even after the successful opening of the vessel [33]. For instance, the risk of angiographically visible distal embolization seems higher in patients with DM [33]. However, the association between DM and no-reflow is less clear. Only a trend toward an increased risk of no-reflow was observed in patients with DM after adjusting for procedural and clinical variables. Additionally, patients with DM experienced longer delays to the first medical contact and reperfusion [2,3,4,33]. Importantly, delayed reperfusion is frequently associated with less frequent TIMI grade 3 flow after PCI and impaired reperfusion on the myocardial level. Myocardial perfusion after PCI is of particular importance, as it has been shown to be a powerful predictor of increased infarct size and worse long-term outcomes of patients with STEMI [34]. Several studies have suggested that patients with DM are less likely to achieve complete myocardial reperfusion assessed with angiography (myocardial blush grade 3 or quantitative myocardial blush) and electrocardiogram (complete ST-segment resolution) after PCI [33,35,36]. On the contrary, a larger analysis from the HORIZONS-AMI trial [5], unlike previous reports, has shown that DM does not affect the achievement of optimal reperfusion after primary PCI. Importantly, successful reperfusion was associated with a decrease in the risk of death by 2/3 in both patients with and without DM, with no interaction between diabetic status and reperfusion success in their effect on 3-year mortality [5]. More recent analysis by Tomasik et al. has confirmed that DM patients with impaired myocardial reperfusion are at higher risk of heart failure and the composite of heart failure and all-cause death at 6 years after STEMI [36]. These findings may suggest that DM worsens outcomes by several mechanisms, not limited to an impairment of reperfusion.

Observed periprocedural mortality was almost two times higher in patients with than without DM. As discussed earlier, successful reperfusion in patients with DM might be delayed due to an atypical presentation frequently observed in diabetic patients [2,3,4,33]. Importantly, ischemia time is a major determinant of survival in patients with STEMI [34,37]. In addition, patients with DM are higher-risk individuals, with advanced age and a high prevalence of comorbidities, for instance, chronic kidney disease and previous stroke [38]. Both are strong predictors of poor outcomes in patients with STEMI. In addition, DM might increase the risk of a more severe course of STEMI, including the development of cardiogenic shock. Cardiogenic shock was obviously identified as the main predictor of periprocedural death in our cohort. Therefore, these factors account for the worse long-term prognosis of diabetic patients presenting with STEMI. On the other hand, DM was not identified as an independent predictor of in-hospital mortality in patients with acute coronary syndrome complicated by cardiogenic shock [39]. More aggressive pharmacotherapy might mitigate these detrimental effects of DM [12,13,14]. Unfortunately, the use of ticagrelor and prasugrel was low but comparable between the patients with and without DM. On the other hand, observed differences in using antiplatelet/antithrombotic agents might be affected by the lack of data on prehospital administration of those agents. Also, the data on other drugs and procedures during the in-hospital stay were not available. Thus, a comprehensive assessment of adherence to recommendations was impossible. A slightly higher rate of puncture site bleeding in patients with DM was observed. However, a joint analysis from the REPLACE-2, ACUITY, and HORIZONS-AMI trials did not confirm an association between diabetic status and the risk of access site bleeding in patients undergoing PCI [40]. Importantly, novel risk scores designed to predict bleeding after PCI did not identify the predictive value of DM [41,42].

We should acknowledge several limitations of this study. Follow-up was limited to the periprocedural period; thus, the assessment of the impact of DM on long-term outcomes was not possible. The interpretation of TIMI flow and no-reflow was based on the operator’s, but not independent core, laboratory assessments. No data on thrombus grading and myocardial blush grading were provided. In addition, no Syntax score calculations were gathered in the registry. No hemoglobin A1c concentrations at admission, nor fasting blood glucose levels during hospital stay/at discharge to confirm the presence of unrecognized DM were available. Data on the type of DM (type 1 or 2), duration of the symptoms, and type/dose of oral hypoglycemic drugs were not collected.

## 5. Conclusions

Despite continuous progress in STEMI treatment, DM remains a strong predictor of periprocedural mortality. However, this detrimental effect of DM may be partially explained by the overall higher risk profile of diabetic patients.

## Figures and Tables

**Table 1:** Baseline characteristics. Values are presented as percentages or median (interquartile range). Abbreviations: CABG = coronary artery bypass grafting; COPD = chronic obstructive pulmonary disease; FMC = first medical contact; MI = myocardial infarction; PCI = percutaneous coronary intervention.

Variable	Diabetes Mellitus		*p* Value
	No (n = 124,422)	Yes (n = 26,360)	
Age [years]	64.0 (56.0, 72.0)	68.0 (61.0, 77.0)	<0.0001
Men	69.4%	57.8%	<0.0001
Arterial hypertension	53.2%	81.1%	<0.0001
Current smoker	30.6%	22.9%	<0.0001
Chronic kidney disease	2.2%	9.0%	<0.0001
Previous stroke	2.5%	6.5%	<0.0001
Previous MI	11.0%	19.8%	<0.0001
Previous PCI	10.5%	17.2%	<0.0001
Previous CABG	1.5%	3.2%	<0.0001
COPD	1.5%	3.1%	<0.0001
Killip class IV on admission	3.7%	5.2%	<0.0001
Direct transfer	24.0%	25.5%	<0.0001
Cardiac arrest	5.2%	4.7%	0.0005
Hypothermia	0.2%	0.2%	0.50
Time delays for patients with symptoms < 24 h			
from pain onset to FMC [min]	120.0 (60.0, 240.0)	127.00 (60.0, 300.0)	<0.0001
from pain onset to inflation or angio [min]	212.0 (134.0, 390.0)	245.00 (150.0, 459.0)	<0.0001
from FMC to inflation or angio [min]	82.0 (56.0, 131.0)	90.00 (60.0, 150.0)	<0.0001

**Table 2:** Procedural technique and pharmacotherapy. Values are presented as percentages or median (interquartile range). Abbreviations: IRA = infarct-related artery; LAD = left anterior descending artery.

Variable	Diabetes Mellitus		*p* Value
	No (n = 124,422)	Yes (n = 26,360)	
Access site			<0.0001
Femoral	25.1%	28.4%	
Radial	74.4%	70.8%	
Other	0.5%	0.8%	
Single-vessel disease	45.1%	34.9%	<0.0001
Bifurcation lesion	7.0%	7.9%	<0.0001
LAD as IRA	40.8%	41.7%	0.016
Aspiration thrombectomy	12.8%	11.8%	<0.0001
Rotablation	0.1%	0.1%	0.034
Implanted stent	92.00%	89.7%	<0.0001
≥2 stents	15.7%	17.9%	<0.0001
Intravascular ultrasound	0.6%	0.6%	0.25
Optical coherence tomography	0.1%	0.1%	0.15
Contrast load [ml]	150.0 (120.0, 200.0)	160.0 (120.0, 200.0)	<0.0001
Radiation dose [mGy]	675.0 (371.0, 1181.0)	820.0 (464.0, 1407.0)	<0.0001
Periprocedural pharmacotherapy			
Aspirin	77.2%	81.2%	<0.0001
P2Y_12_ inhibitor			<0.0001
Clopidogrel	60.9%	64.2%	
Prasugrel	1.3%	1.3%	
Ticagrelor	20.4%	19.6%	
None	17.4%	14.9%	
Glycoprotein IIb/IIIa inhibitor	23.2%	22.2%	0.0003
Unfractionated heparin	83.9%	87.6%	<0.0001
Bivalirudin	0.6%	0.6%	0.21

**Table 3:** Multivariate logistic regression analysis for TIMI grade 0 to 1 at baseline, TIMI 3 after PCI, periprocedural death, no reflow, and any complications.

	TIMI 0 to 1 at Baseline		TIMI 3 after PCI		Periprocedural Death		No Reflow		Any Complications	
Variable	OR (95% CI)	*p* Value	OR (95% CI)	*p* Value	OR (95% CI)	*p* Value	OR (95% CI)	*p* Value	OR (95% CI)	*p* Value
Diabetes mellitus	0.97 (0.93–0.97)	0.08	0.82 (0.77–0.87)	<0.0001	1.36 (1.17–1.58)	<0.0001	1.12 (0.99–1.12)	0.08	1.13 (1.05–1.23)	0.0025
Female gender									1.17 (1.09–1.26)	<0.0001
Previous stroke			0.76 (0.68–0.85)	<0.0001	1.41 (1.10–1.81)	0.0060	1.21 (0.96–1.52)	0.1007	1.27 (1.10–1.48)	0.0013
Previous MI					1.30 (1.05–1.60)	0.0141				
Previous PCI	0.95 (0.91–0.95)	0.0378					0.81 (0.69–0.96)	0.0133		
Previous CABG	0.74 (0.66–0.83)	<0.0001								
Current smoker	0.95 (0.92–0.99)	0.0041	1.21 (1.14–1.29)	<0.0001	0.77 (0.66–0.91)	0.0020				
Arterial hypertension			1.14 (1.08–1.20)	<0.0001	0.68 (0.59–0.77)	<0.0001	1.28 (1.14–1.43)	<0.0001		
Chronic kidney disease	0.88 (0.81–0.95)	0.0020	0.71 (0.64–0.80)	<0.0001			1.92 (1.59–2.32)	<0.0001	1.52 (1.33–1.73)	<0.0001
COPD							1.50 (1.14–1.97)	0.0042		
Cardiac arrest at baseline			0.75 (0.68–0.83)	<0.0001	1.89 (1.61–2.23)	<0.0001			1.70 (1.52–1.89)	<0.0001
Aspiration thrombectomy			0.76 (0.71–0.81)	<0.0001			2.32 (2.06–2.61)	<0.0001	1.78 (1.64–1.94)	<0.0001
Age [per 1 year]			0.98 (0.98–0.98)	<0.0001	1.03 (1.02–1.03)	<0.0001	1.02 (1.02–1.03)	<0.0001	1.02 (1.02–1.02)	<0.0001
Time pain to FMC [per 100 min]	1.15 (1.14–1.17)	<0.0001	0.98 (0.96–1.00)	0.0491	1.10 (1.04–1.16)	0.0013	1.06 (1.05–1.08)	<0.0001	1.04 (1.03–1.05)	<0.0001
Time pain to angiogram [per 100 min]	0.89 (0.88–0.90)	<0.0001	0.94 (0.93–0.96)	<0.0001						
Killip class IV	1.70 (1.55–1.86)	<0.0001	0.37 (0.34–0.40)	<0.0001	8.77 (7.56–10.18)	<0.0001	2.92 (2.48–3.45)	<0.0001	4.87 (4.41–5.38)	<0.0001
Radial approach	0.74 (0.71–0.77)	<0.0001	1.40 (1.33–1.47)	<0.0001	0.35 (0.30–0.40)	<0.0001	0.87 (0.78–0.98)	0.0200	0.62 (0.58–0.67)	<0.0001
Single vessel disease	1.19 (1.16–1.23)	<0.0001	1.11 (1.05–1.16)	<0.0001	0.33 (0.28–0.39)	<0.0001	0.78 (0.70–0.87)	<0.0001	0.63 (0.58–0.67)	<0.0001
LAD as IRA	0.78 (0.76–0.81)	<0.0001	0.89 (0.84–0.93)	<0.0001	1.79 (1.58–2.03)	<0.0001	1.18 (1.06–1.31)	0.0017	1.23 (1.16–1.2)	<0.0001

Values are presented as odds ratios (OR) with 95% confidence intervals (95% CI). Abbreviations: CABG = coronary artery bypass grafting; COPD = chronic obstructive pulmonary disease; FMC = first medical contact; IRA = infarct-related artery; LAD = left anterior descending artery; MI = myocardial infarction; PCI = percutaneous coronary intervention; TIMI = Thrombolysis in Myocardial Infarction.

## Data Availability

The data presented in this study are available on request from the corresponding author.
